# Switching adult patients with spasticity from onabotulinumtoxinA to abobotulinumtoxinA: a real-world data analysis across three US-based treatment centers

**DOI:** 10.57264/cer-2025-0181

**Published:** 2026-05-01

**Authors:** Nate Way, Edward Dabrowski, Mitchell Paulin, Martin Taylor, John Madden, Amandeep Mann, Jonathan Bouchard

**Affiliations:** 1 Real World Evidence, Cerner Enviza, an Oracle Company, Malvern, PA 19301, USA; 2 Beaumont Health, Royal Oak, MI 48073, USA; 3 The Center for Tone Management of the Main Line, Paoli, PA 19301, USA; 4 OrthoNeuro, New Albany, OH 43054, USA; 5 Ipsen, Cambridge, MA 02142, USA

**Keywords:** abobotulinumtoxinA, onabotulinumtoxinA, real-world study, spasticity, treatment switch

## Abstract

**Aim:**

To assess patient characteristics, treatment patterns, botulinum toxin type-A (BoNT-A) costs and describe safety in adults with spasticity who switched from onabotulinumtoxinA to abobotulinumtoxinA.

**Materials & methods:**

Chart data from three US-based treatment centers was collected in patients aged ≥18 years with upper limb (ULS), lower limb (LLS) or ULS and LLS (ULS + LLS) spasticity. Eligible patients had ≥2 onabotulinumtoxinA treatment cycles before switching to abobotulinumtoxinA; they were followed for three additional abobotulinumtoxinA treatment cycles. A *post hoc* analysis of estimated drug costs was conducted.

**Results:**

Eighty-eight patients (mean age 44.9 years; 62.5% male) switched from onabotulinumtoxinA to abobotulinumtoxinA; in 84 (95.5%), the switch was due to ‘medical need/effectiveness not achieved.’ Most common spasticity etiologies were cerebral palsy (43.2%) and stroke (25.0%). Fifty-one patients (58.0%) had bilateral spasticity with a mean ± SD of 5.1 ± 2.2 muscles injected at each visit over the 5-injection-cycle treatment period. No adverse events were reported following switching. Mean estimated cost per visit was $2731 with onabotulinumtoxinA and $1452 with abobotulinumtoxinA.

**Conclusion:**

In this real-world study, patients with ULS, LLS or ULS + LLS who were switched from onabotulinumtoxinA to abobotulinumtoxinA continued for ≥3 treatment cycles without any reported adverse events. Switching resulted in lower estimated BoNT-A costs.

## Plain language summary

**What is this article about?:**

This article explores what happens when adults with spasticity – a condition that causes increased muscle tightness and difficulty with movement – change their treatment. Botulinum toxin type-A (BoNT-A) injections are well established treatments for patients with spasticity. To better understand real-world treatment decisions and outcomes, researchers reviewed the medical records of 88 adult patients treated at three centers in the US. All of these patients had switched from onabotulinumtoxinA (onaBoNT-A) to abobotulinumtoxinA (aboBoNT-A). The study aimed to find out why patients were switched and what happened after the change in treatment.

**What were the results?:**

Most patients (95.5%) were switched because their previous treatment (onaBoNT-A) was not effective enough. After switching to aboBoNT-A, no side effects were reported. Additionally, the estimated cost per treatment visit dropped by approximately 47%.

**What do the results mean?:**

These findings suggest that switching to aboBoNT-A may benefit patients who are not getting enough relief from their current onaBoNT-A treatment. The switch was well-tolerated, and patients needed fewer spasticity-related doctor visits and less rehabilitation therapy afterward. The change in treatment also helped reduce healthcare costs. These real-world results may help doctors and healthcare decision-makers choose the most effective and affordable treatment options for managing spasticity.

Spasticity is a motor disorder characterized by a velocity-dependent increase in muscle tone with exaggerated tendon jerks, resulting from hyper-excitability of stretch reflexes. Spasticity is a common feature of upper motor neuron syndrome commonly associated with nervous system damage such as stroke, traumatic brain injury, cerebral palsy (CP), spinal cord injury or multiple sclerosis [1–3]. It contributes to functional motor disability or impairment, pain and discomfort, affecting patients in different locations, most commonly the upper and/or lower limbs and with varying degrees of severity [1,4]. Goals of spasticity management include improving active and passive range of motion and functional ability, reducing pain and deformity, improving mobility and facilitating concomitant treatments [5].

Spasticity management is often challenging [6] and is primarily focused on physical therapy timing to reduce muscle overactivity and prevent irreversible soft-tissue changes and tendon contractures by maintaining muscle length and normalizing limb posturing [7,8]. Effective management requires a multidisciplinary approach, including consideration of the use of botulinum toxin type-A (BoNT-As) treatments [9]. Furthermore, given the chronic nature of spasticity-related challenges, healthcare costs become an essential variable in management planning [10]. BoNT-As, such as abobotulinumtoxinA (aboBoNT-A) and onabotulinumtoxinA (onaBoNT-A), are well established as first-line treatments for adult spasticity across numerous therapeutic guidelines and can ease symptoms through temporary muscle relaxation [11–13]. When coupled with adjunctive treatments including physical and occupational therapies, BoNT-As can facilitate gains in rehabilitation and goal achievement [14,15].

While BoNT-A products are protein neurotoxins derived from a common bacterium strain of *Clostridium botulinum*, aboBoNT-A and onaBoNT-A each have a unique manufacturing process, different excipients and noninterchangeable potency units [16,17]. Moreover, there are differences in the amounts of the 150 KDa core associated with the total recommended unit doses for each formulation and is the key component of the neurotoxin protein complex mediating BoNT-As therapeutic efforts [16,18]. Collectively, such differences may differentially impact clinical outcomes.

Progress in rehabilitation requires periodic adjustments in treatment, which on occasion include the switching of patients from one BoNT-A formulation to another [19,20]. Data on real-world management of spasticity in both pediatric and adult populations are emerging [21–24]. Retrospective analyses of considerations for switching formulations in these populations indicate that switching from onaBoNT-A to aboBoNT-A is generally well-tolerated, with comparable or improved clinical outcomes and reduced treatment costs [11,21–26].

The current study is a retrospective chart review that expands our understanding of switching formulations in spasticity management. The aim of this preliminary investigation is to contribute to the growing body of evidence by addressing considerations for switching due to treatment formulation failure in adults presenting with spasticity. Here we report on patient characteristics, treatment patterns, preliminary therapeutic findings along with economic consequences (costs considerations) and describe safety in the context of switching patients from onaBoNT-A to aboBoNT-A therapy. These retrospective data were provided by three US centers that have well established experience with BoNT-A usage in adults with spasticity.

## Materials & methods

### Study sample

This was a retrospective, observational study that was conducted using medical chart data from three US-based treatment centers. The study was conducted in accordance with the International Society for Pharmacoepidemiology Guidelines for Good Pharmacoepidemiology Practices, the Declaration of Helsinki and applicable regulatory requirements, as well as with scientific purpose, value and rigor. The study protocol was reviewed and approved by a central institutional review board (Pearl IRB, IN, USA) and was reported in accordance with the Strengthening the Reporting of Observational Studies in Epidemiology (STROBE) guidelines.

Physicians from three experienced centers managing adult spasticity patients with BoNT-As, were recruited to participate in the online survey to collect practice characteristics. Prior to beginning the survey, physicians were provided with a statement of informed consent, which included information about the goals of the study and approximate length of the survey. Relevant data collected as part of routine medical care were entered by participating physicians into an electronic case report form. Patient data were pseudonymized, and the patients were identified by a numeric ID code throughout the research process. To avoid bias in patient selection, physicians were asked to include all consecutive patients meeting eligibility criteria during a defined period between 1 September 2015 and 30 September 2020. For quality assurance before the full launch, a soft launch of the electronic case report form was tested to check for data quality and program functionality.

### Eligibility criteria

Identified physicians needed to have practiced medicine in direct patient care at an established healthcare facility managing adult spasticity in the United States and be able to provide a sufficient (undefined) number of patient records.

Eligible patients were adults (aged ≥18 years) at the time of the chart review, with a diagnosis of upper limb spasticity (ULS), lower limb spasticity (LLS) or both upper and lower limb spasticity (ULS + LLS), who had received at least two onaBoNT-A treatment cycles before switching to aboBoNT-A for at least three subsequent injections. Exclusion criteria were treatments and assessments not having been performed by the same physician or the same facility, major limitations in the passive range of motion in a paretic limb (Modified Ashworth Scale [MAS] score 4 in ≥1 joint), received BoNT-A injection for concomitant conditions (e.g., pain and cosmetic smoothing) or received aboBoNT-A for off-label indications.

### Statistical analyses

Because this was a descriptive study, no formal inferential statistical testing was performed. All analyses were conducted for the aggregate study sample, as well as for the three patient cohorts (ULS, LLS and ULS + LLS). A count of missing data was provided for variables, as appropriate. Means, medians, standard deviations (SDs), minimum values, maximum values and quartiles were reported for all continuous variables. Frequencies and percentages were provided for all categorical variables. Denominators used to calculate percentages were specified for each results table.

A *post hoc* analysis of estimated drug costs was conducted in order to understand the economic consequences of switching patients from onaBoNT-A to aboBoNT-A. Costs were calculated for each dose of onaBoNT-A and aboBoNT-A administered. Estimations were based on the wholesale acquisition costs for the minimum number of vials required and included the potential drug wastage incurred due to discarding of unused portions of vials from either product.

## Results

### Demographics & baseline characteristics

A total of 92 patients were included in the aggregate cohort; 88 patients (95.7%) received onaBoNT-A injections and four patients (4.3%) received injections with incobotulinumtoxinA (incoBoNT-A) before switching to aboBoNT-A at their third visit. The small sample size of patients who were treated with incoBoNT-A before switching to aboBoNT-A prevented any relevant interpretations from being made, and as such, these four patients were excluded from the results presented here.

Of the 88 patients who switched from onaBoNT-A to aboBoNT-A, 62.5% were male, and the overall mean (SD) age was 41.0 (20.0) years, (median 42.5 years) (Table 1). The mean (SD) age at diagnosis of the underlying neurological condition was 25.7 (24.1) years, (median 22.9 years). The mean (SD) time from neurological diagnosis to spasticity diagnosis was 1.9 (6.1) and 1.5 (4.6) years for ULS and LLS, respectively (median 0.07 years for both). Seventeen patients (19.3%) were diagnosed with ULS only, 42 (47.7%) with LLS only and 29 (33.0%) with both types of limb spasticity (ULS + LLS). The most common spasticity etiology was CP (n = 38 [43.2%]). Other etiologies included stroke (n = 22 [25.0%]), traumatic brain injury (n = 12 [13.6%]), multiple sclerosis (n = 8 [9.1%]) and spinal cord injury (n = 2 [2.3%]).

**Table 1.** Patient demographics and characteristics.

	ULS (n = 17)	LLS (n = 42)	ULS + LLS (n = 29)	Total (N = 88)
**Age at visit 1**, (years) Mean (SD) Median	40.5 (19.7) 45.0	36.6 (19.1) 33.5	47.6 (20.2) 56.0	41.0 (20.0) 42.5
**Gender**, n (%) Male Female	9 (52.9%) 8 (47.1%)	27 (64.3%) 15 (35.7%)	19 (65.5%) 10 (34.5%)	55 (62.5%) 33 (37.5%)
**Neurological condition that led to spasticity diagnosis**^†^**, n (%)** Cerebral palsy Stroke Traumatic brain injury Multiple sclerosis Other^‡^ Spinal cord injury	6 (35.3%) 6 (35.3%) 3 (17.6%) – 2 (11.8%) –	24 (57.1%) 1 (2.4%) 4 (9.5%) 7 (16.7%) 4 (9.5%) 2 (4.8%)	8 (27.6%) 15 (51.7%) 5 (17.2%) 1 (3.4%) – –	38 (43.2%) 22 (25.0%) 12 (13.6%) 8 (9.1%) 6 (6.8%) 2 (2.3%)
**Age at diagnosis of neurological condition**, (years) Mean (SD) Median	26.3 (27.1) 14.0	21.3 (22.7) 14.0	31.8 (23.8) 27.0	25.7 (24.1) 22.9
**Time from neurological condition diagnosis to limb spasticity diagnosis, (years)** Mean (SD) Neurological condition → ULS Neurological condition → LLS Median (Q1, Q3) Neurological condition → ULS Neurological condition → LLS	1.9 (6.9) – 0.04 (0.01, 0.06) –	– 1.1 (3.5) – 0.04 (0.0, 0.08)	1.8 (5.8) 2.1 (6.0) 0.08 (0.02, 0.54) 0.08 (0.02, 0.54)	1.9 (6.1) 1.5 (4.6) 0.07 (0.01, 0.26) 0.07 (0.01, 0.26)

^†^There are some inconsistencies in the reporting of cerebral palsy for these questions. The results shown represent the responses of the researchers.

^‡^Specified encephalopathy, Friedreich’s ataxia, hereditary spastic paraparesis and leukodystrophy.

LLS: Lower limb spasticity; SD: Standard deviation; ULS: Upper limb spasticity; ULS + LLS: Upper limb and lower limb spasticity.

### Assessments & techniques

To inform treatment decisions, most patients (97.7%) were assessed at each visit throughout the treatment course (Table 2). Across all injection visits (N = 440), the MAS and/or the Goal Attainment Scale (GAS) were the most frequently used clinical function assessments (used across 61.6% and 59.1% of visits, respectively), regardless of spasticity type. Overall, palpation was predominantly employed as a guided administration technique (59.1% of all visits), followed by electromyography (EMG; 40.9% of all visits); however, in the subgroup of patients with ULS + LLS, EMG was most frequently used (58.6% of all visits). No differences in type of guidance techniques utilized were seen before and after the switch to aboBoNT-A.

**Table 2.** Utilization of assessment scales and techniques across all injection visits.

	ULS (n = 85)	LLS (n = 210)	ULS + LLS (n = 145)	Total (N = 440^†^)
**Assessment(s) across all visits**^‡^**, n (%)** Modified Ashworth Scale (MAS) Goal Attainment Scale (GAS) Other (not specified) Missing (unknown) Clinical Global Impression (CGI) Tardieu Scale (TS) Pain Visual Analogue Scale (VAS) Associated Reaction Rating Scale (ARRS) Arm Activity Measure (ArmA) Functional Ambulation Category (FAC)	85 (100.0%) 65 (76.5%) 55 (64.7%) 20 (23.5%) – – – – – – –	200 (95.2%) 145 (69.0%) 145 (69.0%) 55 (26.2%) 10 (4.8%) – – – – – –	145 (100.0%) 61 (42.1%) 60 (41.4%) 84 (57.9%) – – – – – – –	430 (97.7%) 271 (61.6%) 260 (59.1%) 159 (36.1%) 10 (2.3%) – – – – – –
**Guided administration techniques used across all injection procedures, n (%)** Palpation EMG Ultrasound None Missing	55 (64.7%) 30 (35.3%) – – –	145 (69.0%) 65 (31.0%) – – –	60 (41.4%) 85 (58.6%) – – –	260 (59.1%) 180 (40.9%) – – –

^†^N was computed based on each patient having multiple visits.

^‡^This was a multi-select question; researchers could select all responses that applied.

EMG: Electromyography; LLS: Lower limb spasticity; ULS: Upper limb spasticity; ULS + LLS: Upper limb and lower limb spasticity.

### BoNT-A treatment & injection intervals

Overall, bilateral spasticity was more prevalent (n = 51; 58.0%) than unilateral spasticity among patients who had switched BoNT-A therapies, driven by those in the LLS cohort (n = 37/42; 88.1%) (Table 3). Injections were evenly distributed between the right and left lower limbs and were administered more frequently to the lower limbs (61.4–63.6% left-lower; 64.8–65.9% right-lower) than to the upper limbs (28.4–30.7% left-upper; 31.8–35.2% right-upper).

**Table 3.** Dose and injection intervals.

	ULS (n = 17)	LLS (n = 42)	ULS + LLS (n = 29)	Total (N = 88)
**Unilateral spasticity, n (%)** Overall study duration (visits 1–5)	11 (64.7%)	5 (11.9%)	21 (72.4%)	37 (42.0%)
**Bilateral spasticity, n (%)** Overall study duration (visits 1–5)	6 (35.3%)	37 (88.1%)	8 (27.6%)	51 (58.0%)
**Injection intervals, (weeks)**				
Visits 1–2 Mean (SD) Median (Q1, Q3)	25.4 (26.0) 15.4 (14.1, 19.0)	18.3 (9.3) 15.0 (14.0, 17.0)	17.7 (9.1) 15.0 (14.0, 16.7)	19.5 (14.2) 15.0 (14.0, 17.0)
Visits 2–3 Mean (SD) Median (Q1, Q3)	19.3 (10.1) 15.0 (14.0, 21.6)	21.7 (16.3) 15.0 (14.00, 19.7)	23.3 (15.8) 16.0 (14.7, 24.0)	21.8 (15.0) 15.2 (14.0, 21.7)
Visits 3–4 Mean (SD) Median (Q1, Q3)	26.1 (26.5) 14.0 (14.0, 17.0)	14.9 (1.5) 14.7 (14.0, 15.0)	17.8 (6.1) 15.0 (14.0, 20.0)	18.0 (12.7) 14.5 (14.0, 16.5)
Visits 4–5 Mean (SD) Median (Q1, Q3)	22.4 (20.3) 14.1 (14.0, 21.6)	19.0 (11.6) 15.0 (14.0, 18.0)	16.3 (3.9) 15.0 (14.0, 17.0)	18.7 (12.2) 15.0 (14.0, 17.9)
**Overall injection intervals, (visits 1–5)** Mean (SD) Median (Q1, Q3)	23.2 (13.1) 16.3 (15.4, 33.8)	18.4 (5.7) 16.0 (14.4, 20.3)	19.2 (5.9) 17.3 (14.9, 20.6)	19.6 (7.8) 16.3 (14.7, 20.4)
**Total dosage (units), mean (SD)**				
Visit 1	408.8 (185.6)	458.3 (144.8)	447.8 (160.6)	445.3 (157.7)
Visit 2	441.2 (180.5)	495.8 (129.0)	460.7 (155.7)	473.7 (148.8)
Visit 3	876.4 (414.4)	934.9 (374.7)	1190.3 (316.3)	1009.5 (383.6)
Visit 4	1055.6 (475.6)	1023.3 (357.8)	1222.6 (356.6)	1096.7 (389.3)
Visit 5	1083.3 (461.8)	1060.5 (384.9)	1196.8 (400.4)	1110.9 (406.1)
Visits 1–2	425.0 (179.0)	477.1 (132.8)	454.2 (156.0)	459.5 (149.7)
Visits 3–5	985.8 (444.7)	1011.1 (336.7)	1211.5 (334.8)	1072.3 (368.2)
**Muscles injected, n, mean (SD)**				
Visit 1 Min, max	5.5 (2.2) 3.0, 12.0	3.8 (1.9) 1.0, 8.0	5.7 (2.1) 2.0, 10.0	4.8 (2.2) 1.0, 12.0
Visit 2 Min, max	5.7 (2.5) 2.0, 12.0	3.9 (2.0) 1.0, 8.0	6.0 (2.1) 2.0, 10.0	4.9 (2.3) 1.0, 12.0
Visit 3 Min, max	5.5 (2.3) 2.0, 12.0	4.1 (2.0) 2.0, 8.0	6.3 (2.3) 2.0, 13.0	5.1 (2.3) 2.0, 13.0
Visit 4 Min, max	6.0 (2.4) 2.0, 12.0	4.2 (1.9) 2.0, 8.0	6.4 (2.5) 2.0, 12.0	5.3 (2.4) 2.0, 12.0
Visit 5 Min, max	6.1 (2.4) 2.0, 12.0	4.1 (1.9) 2.0, 8.0	6.4 (2.4) 2.0, 12.0	5.3 (2.4) 2.0, 12.0
Visits 1–2 Min, max	5.6 (2.3) 3.0, 12.0	3.9 (1.9) 1.0, 8.0	5.9 (2.1) 2.0, 10.0	4.8 (2.2) 1.0, 12.0
Visits 3–5 Min, max	5.9 (2.4) 2.0, 12.0	4.1 (1.8) 2.0, 8.0	6.4 (2.2) 2.7, 12.3	5.2 (2.3) 2.0, 12.3
Overall study duration (visits 1–5) Min, max	5.7 (2.3) 2.4, 12.0	4.0 (1.8) 2.0, 8.0	6.2 (2.0) 2.4, 11.4	5.1 (2.2) 2.0, 12.0
**Time of follow-up (weeks), mean (SD)**				
Overall study duration (visits 1–5)	94.4 (53.3)	73.9 (22.9)	75.5 (22.9)	78.4 (31.5)

LLS: Lower limb spasticity; Max: Maximum; Min: Minimum, SD: Standard deviation; ULS: Upper limb spasticity; ULS + LLS: Upper limb and lower limb spasticity.

Across all five visits, between 52.3 and 62.5% of patients had their visits scheduled automatically. The mean (SD) interval between the first 2 onaBoNT-A injections was 19.5 (14.2) weeks (median 15.0 weeks) (Table 3). The average treatment interval ranged from 17.7 (9.1) weeks (median 15.0 weeks) for patients with ULS + LLS to 25.4 (26.0) weeks (median 15.4 weeks) for patients with ULS only. The mean (SD) dose of onaBoNT-A administered was 425.0 (179.0) U, 477.1 (132.8) U, and 454.2 (156.0) U in patients with ULS, LLS and ULS + LLS, respectively.

After switching to aboBoNT-A treatment, the mean (SD) interval was 18.0 (12.7) weeks (median 14.5 weeks) between visits 3 and 4 and 18.7 (12.2) weeks (median 15.0 weeks) between visits 4 and 5 (Table 3). The injection intervals were comparable between visits 3 and 4 and visits 4 and 5. The mean (SD) aboBoNT-A doses across three treatment cycles were 985.8 (444.7) U, 1011.1 (336.7) U and 1211.5 (334.8) U for patients with ULS, LLS and ULS + LLS, respectively.

Throughout the treatment course, the average number of muscles injected with any BoNT-A therapy was 5.1 (2.2) (Table 3). During visits 1 and 2, the average number of muscles injected was 4.8 (2.2), increasing to 5.2 (2.3) for visits 3–5.

### Healthcare resource utilization & costs

Between injection-related visits, a vast majority of patients (87.5%) had office visits related to their spasticity diagnosis without receiving BoNT-A injections (Table 4). The frequency of spasticity-related office visits without BoNT-A injections increased overall, from 45.5% between visits 1–2 to 69.3% between visits 2–3. After switching to aboBoNT-A therapy, a reduction in spasticity-related office visits was observed between visits 3 and 4 (to 55.7%) and further between visits 4 and 5 (to 35.2%). Compared with other spasticity groups, patients diagnosed with ULS + LLS were more likely to experience spasticity-related office visits without injections; these same patients also had the greatest reduction in healthcare resource utilization after switching to aboBoNT-A therapy.

**Table 4.** Healthcare utilization during study period across five injection cycles.

	ULS (n = 17)	LLS (n = 42)	ULS + LLS (n = 29)	Total (N = 88)
**Healthcare resource use between any visit**^^†^^, n (%) Office visit related to spasticity (no BoNT-A injection given) Prescription medication(s) None Office visit related to other issues unrelated to spasticity Hospitalization related to other issues unrelated to spasticity ER visit related to other issues unrelated to spasticity ER visit related to spasticity Hospitalization related to spasticity	12 (70.6%) 8 (47.1%) 4 (23.5%) 4 (23.5%) – – – –	36 (85.7%) 17 (40.5%) 6 (14.3%) 3 (7.1%) 1 (2.4%) – – –	29 (100.0%) 5 (17.2%) – 1 (3.4%) 2 (6.9%) 1 (3.4%) – –	77 (87.5%) 30 (34.1%) 10 (11.4%) 8 (9.1%) 3 (3.4%) 1 (1.1%) – –
**Other medication(s) taken by the patient at any visit**^^†^^, n (%) Systemic antispasticity medications (e.g., baclofen, tizanidine and dantrolene) Phenol, alcohol, or other neurolytic agents Pain medications for simple pain relief (e.g., paracetamol and NSAIDs) None Pain medications for neuropathic pain and spasticity (e.g., gabapentin, pregabalin and amitriptyline) Other (specified ITB, lumbar injection and right tibial nerve block) Opioids	11 (64.7%) 5 (29.4%) 7 (41.2%) 4 (23.5%) – – –	24 (57.1%) 5 (11.9%) 7 (16.7%) 12 (28.6%) 3 (7.1%) – 3 (7.1%)	24 (82.8%) 16 (55.2%) 5 (17.2%) 2 (6.9%) 2 (6.9%) 1 (3.4%) 1 (3.4%)	59 (67.0%) 26 (29.5%) 19 (21.6%) 18 (20.5%) 5 (5.7%) 4 (4.5%) 1 (1.1%)
**Ancillary therapies utilized by the patient at any visit**^^†^^, n (%) Physical therapy Occupational therapy None Splint Other (specified AxioBionic sleeves) Surgery	11 (64.7%) 11 (64.7%) 4 (23.5%) 1 (5.9%) – –	31 (73.8%) 20 (47.6%) 10 (23.8%) 1 (2.4%) – –	26 (89.7%) 22 (75.9%) 3 (10.3%) 1 (3.4%) 1 (3.4%) –	68 (77.3%) 53 (60.2%) 17 (19.3%) 3 (3.4%) 1 (1.1%) –

^†^This was a multi-select question; researchers could select all responses that applied.

BoNT-A: Botulinum toxin type-A; ER: Emergency room; ITB: Intrathecal baclofen pump; LLS: Lower limb spasticity; NSAID: Nonsteroidal anti-inflammatory drug; ULS: Upper limb spasticity; ULS + LLS: Upper limb and lower limb spasticity.

More than a third (34.1%) of patients used prescription medication(s) to manage their illness (Table 4). At least one concomitant medication was utilized by 79.6% of patients to treat their spasticity during the treatment period, with the most common being systemic anti-spasticity drugs (67.0%). The proportion of patients taking systemic anti-spasticity medication was 67.0% at visits 1 and 2, and 65.9% at visits 3–5. Concomitant physical and occupational therapies were used by 77.3% and 60.2% of patients, respectively, to manage their illness (Table 4). The overall use of physical and occupational therapies decreased throughout the treatment course (physical therapy: visit 1: 71.6%, visit 2: 65.9%, visit 3: 68.2%, visit 4:62.5%, visit 5: 64.8%; occupational therapy: visit 1: 56.8%, visit 2: 52.3%, visit 3: 52.3%, visit 4: 46.6%, visit 5: 45.5%).

**Figure 1.**
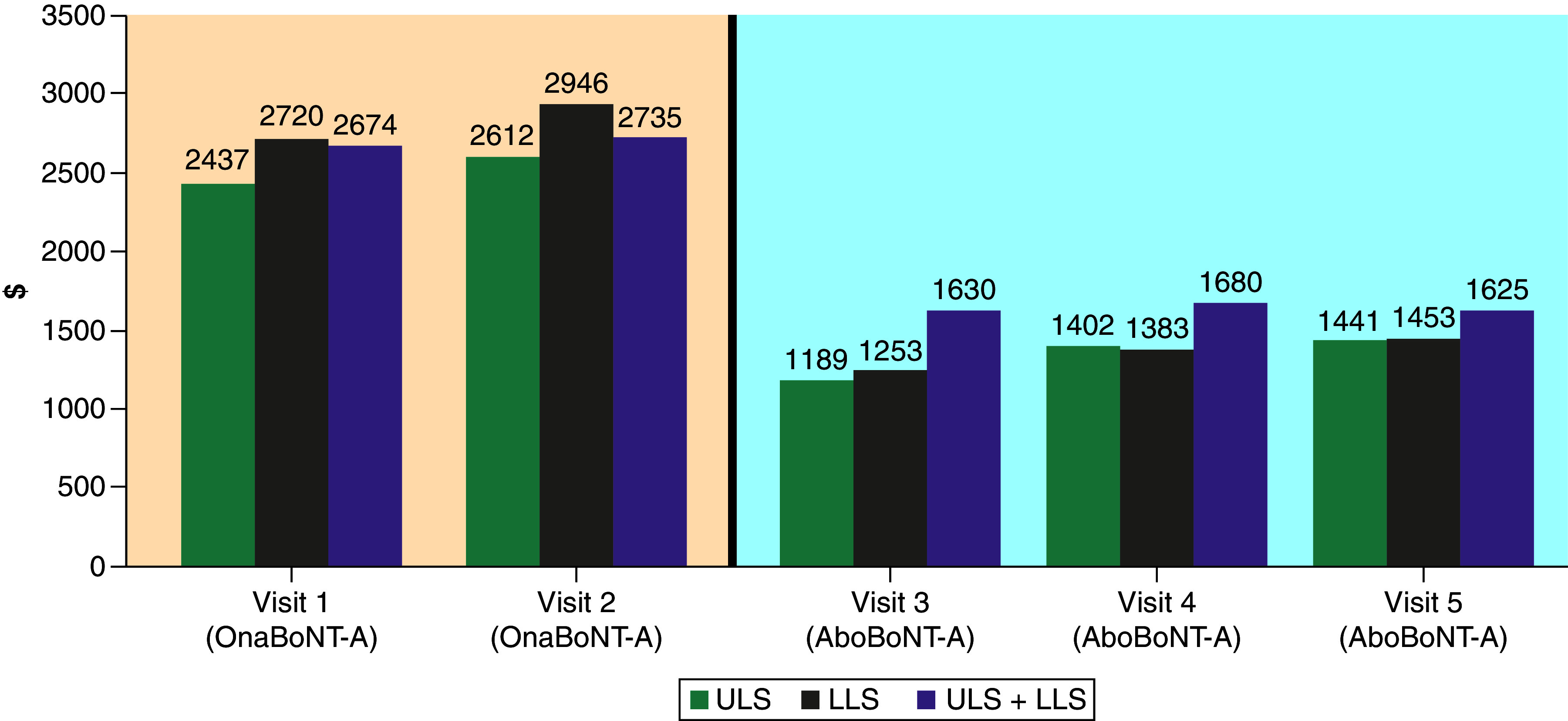
Mean BoNT-A costs by injection visit. AboBoNT-A: AbobotulinumtoxinA; BoNT-A: Botulinum toxin type-A; LLS: Lower limb spasticity; OnaBoNT-A: OnabotulinumtoxinA; ULS: Upper limb spasticity; ULS + LLS: Upper limb and lower limb spasticity. Light orange: OnaBoNT-A; Light blue: AboBoNT-A.

*Post hoc* analysis of cost data showed that patients incurred considerably higher mean drug costs per visit during visits 1 and 2 than they did during visits 3–5, especially patients with LLS (Table 5 & Figure 1). Switching patients from onaBoNT-A to aboBoNT-A therapy resulted in lower estimated drug costs across all spasticity types, amounting to a 46.8% reduction overall.

**Table 5.** Mean overall BoNT-A costs.

Spasticity type	OnaBoNT-A visit 1	OnaBoNT-A visit 2	Mean OnaBoNT-A cost (visits 1 + 2)	AboBoNT-A visit 3	AboBoNT-A visit 4	AboBoNT-A visit 5	Mean AboBoNT-A cost (visits 3 + 4 + 5)
ULS	$2437	$2612	$2524	$1189	$1402	$1441	$1334
LLS	$2720	$2946	$2833	$1253	$1383	$1453	$1363
ULS + LLS	$2674	$2735	$2705	$1630	$1680	$1625	$1645
Overall mean	$2650	$2812	$2731	$1365	$1485	$1508	$1452

AboBoNT-A: AbobotulinumtoxinA; BoNT-A: Botulinum toxin type-A; LLS: Lower limb spasticity; Max: Maximum; Min: Minimum, OnaBoNT-A: OnabotulinumtoxinA, SD: Standard deviation; ULS: Upper limb spasticity; ULS + LLS: Upper limb and lower limb spasticity.

### Reasons for switching BoNT-A preparations

For the majority of patients (n = 84 [95.5%]), the switch to aboBoNT-A therapy was attributed to a lack of effectiveness (‘medical need/effectiveness not achieved’) of initial BoNT-A treatment, which was consistent across all groups (ULS: n = 17/17 [100.0%]; LLS: n = 39/42 [92.9%]; ULS + LLS: n = 28/29 [96.6%]). A small proportion of patients switched because of medical policy changes (n = 2 [2.3%]) or unknown reasons (n = 2 [2.3%]).

### Adverse events & special situations

Among all patients analyzed in this study, neither adverse events nor special situations were reported at any point throughout their treatment with aboBoNT-A.

## Discussion

This retrospective, observational study provides further real-world evidence on the clinical characteristics, treatment patterns and economic outcomes in patients with ULS and/or LLS who switched from onaBoNT-A to aboBoNT-A. Among patients who received at least three treatment cycles of aboBoNT-A following prior treatment with onaBoNT-A, the switch was associated with favorable safety, sustained use and reductions in spasticity-related office visits and estimated drug costs.

These findings are consistent with previously published real-world analyses describing treatment patterns following switching from onaBoNT-A to aboBoNT-A regarding clinical effectiveness and economic considerations [21–25,27]. Prior retrospective studies have consistently reported that switching from onaBoNT-A to aboBoNT-A is generally well-tolerated with comparable or improved outcomes, often accompanied by reductions in treatment-related costs. Notably, many of these studies describe switching driven primarily by administrative or cost-effectiveness considerations rather than suboptimal efficacy [21–25,27]. In contrast, the present study evaluated switching in the context of insufficient clinical response, offering a distinct perspective on treatment optimization in BoNT-A–treated individuals with spasticity. The absence of adverse events and special situations among all patients analyzed in the current study – at any point throughout the treatment course with aboBoNT-A – further reinforces the favorable safety and tolerability profile of aboBoNT-A in previously BoNT-A–treated populations.

Throughout the treatment course, mean doses of both onaBoNT-A and aboBoNT-A were generally aligned with the upper ranges of their respective dosing guidelines. While treatment dosage is established based on guidelines for specific indications and adjusted according to the size of the muscles and disease severity, real-world doses tend to vary among BoNT-As.

The mean interval between onaBoNT-A injections (visits 1 and 2) was similar to the mean intervals between aboBoNT-A injections (visits 3–5) in the patients who had initially received onaBoNT-A and had switched to aboBoNT-A. Although injection intervals are largely influenced by BoNT-A's duration of effect, various external factors can play a role. Notably, over half of patients had their treatment visits scheduled automatically across the five visits. Since our study did not capture efficacy outcomes consistently at every visit, we cannot reliably compare the relative effectiveness between the two BoNT-A formulations between treatment cycles. Additionally, given the limitations of the study design, we cannot conclude the comparative duration of effect for aboBoNT-A and onaBoNT-A.

In this study, medical need for treatment was the main driver for BoNT-A injections among all eligible patients throughout the treatment course, and symptoms and eligibility were assessed at each visit. The most frequently used tools in this study to assess clinical outcomes were the MAS and GAS instruments (used across 61.6% and 59.1% of visits, respectively), which evaluated treatment need from both the physician’s and the patient’s perspectives. The MAS is a 6-point scale that measures muscle tone by assessing the resistance of the muscle to passive lengthening or stretching, and is widely used to grade severity of muscle spasticity and assess intervention effectiveness [28]. The GAS semi-quantitatively assesses goal attainment and provides information on patients’ priority goals for treatment [29]. It is a 5-point numeric scale ranging from -2 to +2 and scores can be combined in a GAS T-score that is normally distributed around a mean of 50 [29]. Such assessments for determining patients’ treatment needs inform subsequent treatment decisions. Future studies investigating consistent and regular treatment outcomes may thus allow a more granular comparison between BoNT-A treatment paths.

Across all injection visits, guidance techniques observed also reflected real-world practice. Palpation was the most frequently used guidance technique in patients with isolated ULS or LLS, whereas EMG was most frequently used in patients with combined ULS + LLS. Although the use of other guidance techniques – specifically ultrasound [30,31] – has been recently emphasized in the literature, its use was not observed in the present study, which may be attributable to prevailing clinical practices/physician preferences, at the time period during which this chart review was conducted.

For most patients, the switch to aboBoNT-A was attributed to the lack of effectiveness of previous BoNT-A treatment, consistent with published literature. Prior studies have shown that perceived effectiveness of BoNT-A therapy in spasticity management is closely linked to the definition and attainment of individualized treatment goals, underscoring the importance of aligning treatment goals and patient expectations when interpreting clinical response [32–34]. Following the switch to aboBoNT-A, fewer spasticity-related office visits and a significant decrease in estimated drug costs were observed. These findings are aligned with previous studies reporting improved clinical response, enhanced health utility and economic benefit with aboBoNT-A [35]. Moreover, concomitant nonpharmacological therapies were an integral part of the treatment strategy for spasticity, consistent with other studies, that recognize the use of concomitant rehabilitation therapies as a factor that could enhance treatment efficacy [14,15]. Physical and occupational therapies were common early in treatment but declined over successive cycles, a trend documented in other longitudinal cohorts of BoNT-A use [15,32]. This pattern may reflect evolving rehabilitation needs over time as patients' clinical status changed, rather than a reduced role of physical and occupational therapy in spasticity management.

As is inherent with any research relying on sampling methods, the study design had some limitations that should be considered when interpreting the results. Even though special care was taken to avoid bias in recruitment by asking physicians to pull charts sequentially, the actual selection of charts was not supervised. Any inconsistencies observed in the data collected from patient charts could not be verified with the physician respondents retroactively; however, to ensure high electronic case report form validity, the survey was tested pro-actively and logic checks were enforced for validating the accuracy of data collected. Patient charts were selected based on a specific set of inclusion criteria; therefore, data collected may not be representative of all patients with spasticity. Adverse events were recorded as a binary outcome, and the retrospective design and sample size may have limited the detection of rare or subtle events. Estimated drug costs reflected real-world dosing and vial utilization based on wholesale acquisition costs. As no predefined unit conversion ratio was applied, the analysis was not designed to attribute observed cost differences to dose equivalence or individual cost drivers.

## Conclusion

The results of this study are consistent with previous literature and suggest that aboBoNT-A is not only a safe and well-tolerated option but is also cost-effective versus onaBoNT-A treatment for spasticity. Further randomized, controlled, comparative, and switch studies evaluating the efficacy and safety of onaBoNT-A versus aboBoNT-A are warranted. In conclusion, the therapeutic benefit of aboBoNT-A following the switch from onaBoNT-A was demonstrated by the decreased spasticity-related office visits and associated reduced healthcare costs, decreased utilization of rehabilitation therapies and lack of adverse events. These findings suggest that switching can preserve therapeutic benefits and, in some patients, may further enhance them, even when prior treatment efficacy has diminished. By offering a longitudinal view into the therapeutic journey of patients with spasticity treated with available BoNT-A formulations, results from this study make a valuable contribution to the broader understanding of how physicians use BoNT-A therapy to manage various types of limb spasticity in real-world clinical practice. These data also share the rationales underpinning treatment decisions, including switching of BoNT-A formulations. Taken together, the results from this study offer guidance to clinicians and policy makers in managing adult spasticity.

## Summary points

- This retrospective observational study examined real-world patient characteristics, treatment patterns and healthcare utilization in adults with spasticity who switched from onabotulinumtoxinA (onaBoNT-A) to abobotulinumtoxinA (aboBoNT-A).
- For most patients, the switch to aboBoNT-A was prompted by suboptimal effectiveness of onaBoNT-A.
- Nearly all visits (97.7%) included clinical assessments, most commonly using the MAS (61.6%) and GAS (59.1%) to evaluate muscle tone and individualize treatment goals.
- Palpation was the most commonly used injection guidance technique across spasticity types; use of ultrasound guidance, although increasingly recommended, was not observed.
- Mean doses of both onaBoNT-A and aboBoNT-A were generally within the upper range of approved guidelines, and injection intervals were similar across treatment phases.
- Spasticity-related office visits declined following the switch to aboBoNT-A, with the greatest reduction observed in patients with both upper and lower limb spasticity, who had more frequent visits without injections prior to switching.
- Switching from onaBoNT-A to aboBoNT-A led to a substantial reduction in estimated drug costs across all spasticity types, with an overall 46.8% decrease, demonstrating consistent economic benefit regardless of limb involvement.
- Use of physical and occupational therapy declined over the treatment course, consistent with evolving rehabilitation needs in the context of individualized goal setting and attainment during longitudinal BoNT-A therapy.
- No adverse events or special situations were reported at any point during the study, including after patients switched to aboBoNT-A, demonstrating that it is a safe and well-tolerated treatment option in previously BoNT-A–treated patients with spasticity.
- These results offer practical guidance for clinicians and policymakers aiming to optimize treatment strategies and resource allocation for adult spasticity care, supporting the clinical and economic value of switching BoNT-A formulations.
